# Advances in plant polysaccharides in cancer therapy targeting ferroptosis

**DOI:** 10.3389/fphar.2026.1726394

**Published:** 2026-05-01

**Authors:** Yutong Li, Chong Zhou, Ziwei Wang, Wei Ni, Hao Xu, Yingjie Xing, Li Huang

**Affiliations:** 1 Institute of Traditional Chinese and Zhuang-Yao Ethnic Medicine, Guangxi University of Chinese Medicine, Nanning, China; 2 College of Basic Medicine, Guangxi University of Chinese Medicine, Nanning, China

**Keywords:** plant polysaccharide, traditional Chinese medicine, ferroptosis, cancer therapy, review

## Abstract

Cancer is a global health public issue with an increasing morbidity and mortality. Ferroptosis is a form of regulated cell death characterized by iron accumulation and lipid peroxidation, providing a novel and promising strategy for cancer treatment. Plant polysaccharides possess low toxicity, minimal side effects, and significant therapeutic potential, particularly in antioxidation, immune regulation, and cancer suppression. However, it is unknown that whether and how ferroptosis is involved in the anticancer effects of plant polysaccharides. Occurrence of ferroptosis in cancer cells involves with several cell events including disturbed iron metabolism, overwhelmed lipid metabolism, and disordered antioxidant defense, which is fatal to cancer cells. Recent studies have showed that plant polysaccharides play significant roles in cancer treatment with activities in ferroptosis regulation through inhibiting cancer growth, enhancing treatment sensitivity, reducing drug resistance, strengthening immune function. This review presents the current understanding of polysaccharides regulation in cancer treatment targeting ferroptosis, and also address the limitation and future direction of polysaccharide application regarding cancer treatment via ferroptosis. These findings would contribute to advancing cancer therapy.

## Graphical Abstract

**Figure:**
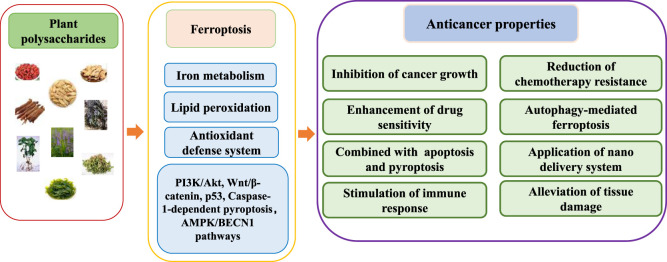

## Highlights

- Plant polysaccharides are natural compounds with promising anticancer effects.
- Plant polysaccharides exert anticancer effects in cancer cells through activation of ferroptosis.
- Plant polysaccharides alleviate noncancer tissue injury through inhibition of ferroptosis.
- Insights into therapeutic application of plant polysaccharides in cancers targeting ferroptosis were explored.

## 1 Introduction

Recently, the increasing incidence and mortality of cancer have become a major threat to human health (Huq et al., 2024). According to estimates by the International Agency for Research on Cancer (IARC), there were nearly 20 million new cancer cases globally in 2022, with cancer-induced deaths reaching 9.7 million cases. By 2050, new cancer cases would be estimated to 35 million (Bray et al., 2024). Although current cancer treatment, including surgery, chemotherapy, radiotherapy, immunotherapy, and targeted therapy, effectively helps to inhibit cancer growth and metastasis, the clinical efficacy of these therapies is often compromised by adverse side effects, high costs, and drug resistance. Therefore, exploring novel cancer-targeted strategies is essential for improvement of cancer treatment.

Ferroptosis is a novel form of iron-dependent programmed cell death, characterized by iron accumulation, lipid peroxidation and antioxidant imbalance, which extremely is distinct from necrosis, apoptosis, and autophagy (Tang et al., 2021; Xie et al., 2016). Morphologically, ferroptotic cells display shrunken mitochondria, increased membrane density, and reduced or absent mitochondrial cristae (Tang et al., 2021). Mechanistically, ferroptosis is triggered by glutathione (GSH) depletion and decreased activity of glutathione peroxidase 4 (GPX4), leading to imbalance between lipid peroxidation, iron metabolism and antioxidant defense system, ultimately driving ferroptotic cell death (Mou et al., 2019). Accumulating evidences demonstrate that ferroptosis plays a pivotal regulatory role in various cancers, including non-small cell lung cancer, breast cancer, hepatocellular carcinoma, colorectal cancer, and ovarian cancer (Li et al., 2020; Zhao et al., 2022). Targeting ferroptosis has been proven a highly promising strategy for cancer therapy, especially drug-resistant cancer.

Traditional Chinese medicine (TCM) has been practiced for a long history and currently is still widely accepted as an alternative treatment for cancer (Zhang et al., 2020). Plants and their metabolite are a key component of TCM. Their multi-target and multi-pathway attributes endow excellent anticancer effects and making them a significant resource for discovery of anticancer drugs. Polysaccharides, one class of plant bioactive metabolites, have been demonstrated to exhibit exceptional structural diversity and a broad range of biological activities, including anticancer, immunomodulatory, and antioxidant effects (Wang et al., 2024c). Notably, plant polysaccharides can regulate various forms of programmed cell death such as apoptosis, necrosis, autophagy, and ferroptosis (Zhang et al., 2023). In addition, nanoparticle formulations of polysaccharides improve therapeutic efficacy by improving drug bioavailability and reducing toxicity (Ren et al., 2022). Interestingly, plant polysaccharides have excellent bidirectional regulatory functions. On one hand, they effectively suppress cancer cell proliferation, on the other hand, they also protect normal cells from the toxicity of anticancer therapies (Liu et al., 2017). Therefore, plant polysaccharides are regarded as promising candidate drugs for cancer treatment (Kubczak et al., 2021).

Emerging evidences have demonstrated that ferroptosis is involved in the regulatory activities of polysaccharides in cancer. Herein, this review aimed to provides a comprehensive insight in the anticancer mechanisms of plant polysaccharides targeting ferroptosis, as well as their roles in alleviating side effects caused by cancer drugs. These findings may contribute to new therapeutic strategies and directions for cancer treatment.

## 2 Methods

### 2.1 Literature search

PubMed, Web of Science, CNKI, Wanfang, and VIP databases were searched, using keywords such as “ferroptosis”, “cancer”, “traditional Chinese medicine” and “polysaccharide” to retrieve studies on the therapeutic potential of plant polysaccharides in cancer treatment targeting ferroptosis.

### 2.2 Inclusion and exclusion criteria

Studies on the treatment of various cancers including breast cancer, esophageal cancer, hepatocellular carcinoma, lung cancer, gastric cancer, ovarian cancer, colorectal cancer, and urothelial carcinoma using plant polysaccharides targeting ferroptosis were included. Additionally, studies investigating plant polysaccharides in reducing cancer drug resistance, enhancing immune responses, and alleviating cardiac, hepatic, and renal toxicities induced by radiotherapy or chemotherapy were included.

Non-experimental studies with polysaccharides extracted from Chinese herbal metabolite prescription, duplicate and incomplete studies, studies with abnormal data or credibility issues, and studies lacking ethical approval were excluded.

### 2.3 Results

Two researchers independently conducted searches using relevant keywords and screened the titles, abstracts, and full texts in accordance with the inclusion and exclusion criteria.

## 3 Mechanisms of ferroptosis in cancer cells

**FIGURE 1:**
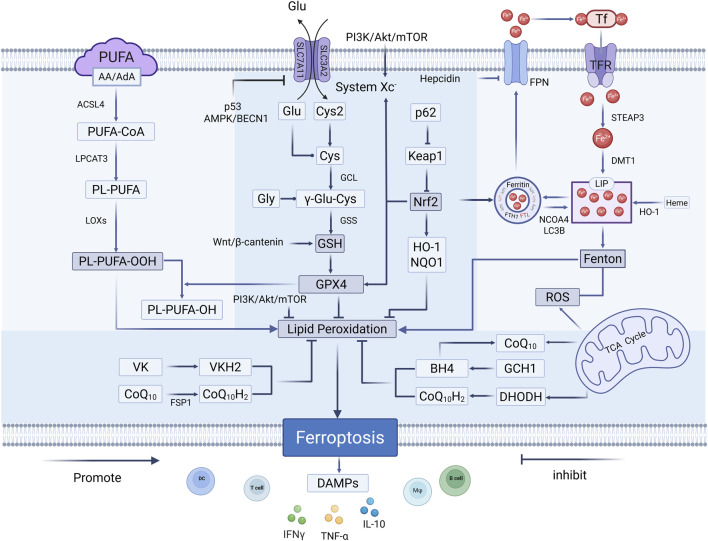
The main mechanism of ferroptosis in cancers. The main signal pathways of ferroptosis include dysregulated iron metabolism, increased lipid peroxidation, and impaired antioxidant defense.

Ferroptosis is tightly associated with iron-dependent lipid peroxidation and involves multiple cellular events, including iron metabolism, lipid peroxidation and antioxidant defense, which has been proven to contribute to modulating initiation and progression of cancer (Brown et al., 2024). It is crucial to outline the core mechanisms of ferroptosis in cancer cells for effectively guiding its application in cancer treatment (Figure 1).

### 3.1 Iron metabolism

Iron is one important determinant for ferroptosis. In an oxygen-rich environment, iron can react with different types of phospholipid hydroperoxides and lipid (fatty acid) hydroperoxides to generate reactive oxygen species (ROS) through the Fenton reaction. Proliferation of cancer cell is highly dependent on iron, and abnormal iron metabolism triggers an increase in susceptibility to ferroptosis (Rodriguez et al., 2022). Specifically, free ferric iron is shuttled into cells bound by transferrin (TF), and reduced to ferrous iron via catalyzing by endosomal six-transmembrane epithelial antigen of prostate 3 (STEAP3), subsequently ferrous iron is released into the cytoplasm through divalent metal transporter 1 (DMT1) to expand the labile iron pool (LIP) (Xu et al., 2024). Most iron is stored in heme or ferrintin. As one antioxidant enzyme, heme oxygenase-1 (HO-1) catalyzes heme to increase accumulation of ferrous iron, ROS production and lipid peroxidation, thereby triggers ferroptosis (Tang et al., 2025). HO-1 is activated by nuclear factor erythroid 2-related factor 2 (Nrf2) under oxidative stress (Chiang et al., 2018). Ferritin, including ferritin heavy chain 1 (FTH1) and ferritin light chain (FTL), sequesters excess intracellular iron (Zhou et al., 2024). Under the catalysis of nuclear receptor coactivator 4 (NCOA4) and microtubule-associated protein 1 light chain 3B (LC3B), large amounts of ferrous iron is released by ferritin through ferritinophagy, and enforce the vulnerability to ferroptosis (Cai et al., 2023; Zhou et al., 2024). Ferroprotein (FPN, also known as SLC40A1) is recognized as the only cellular efflux channel for iron transportation in the cells (Chen et al., 2021; Su et al., 2020). Therefore, the molecules or factors involving in iron metabolism could be vital biomarkers for the regulation of ferroptosis in cancer.

### 3.2 Lipid peroxidation

Lipid peroxidation is one hallmark of ferroptosis, and also the basis for ferroptosis. It is the process mediated by ROS that oxygen free radicals attack unsaturated fatty acids to generate lipids containing peroxy groups (Jia et al., 2023). As prominent phospholipids in cell membranes, PUFAs are particularly susceptible to peroxidation, resulting in disruption of membrane integrity and permeability in cancer cells, ultimately favoring induction of ferroptosis (Endale et al., 2023). Mechanistically, as primary substrates of PUFAs, arachidonic acid (AA) and adrenic acid (AdA) in their free state are catalyzed by acyl-CoA synthase long-chain family member 4 (ACSL4) and lysophosphatidylcholine acyltransferase 3 (LPCAT3) to form PL-PUFA complexes. These PL-PUFAs are then oxidized by iron-dependent enzymes as lipoxygenases (LOXs) or the labile iron pool into lipid hydroperoxides (PL-PUFA-OOH) to undergo the Fenton reaction under the presence of ferrous iron to trigger ferroptosis onset (Lee and Gan, 2022; Xl et al., 2022). Lipid peroxidation by-products, including primary lipid hydroperoxides (LOOH), malondialdehyde (MDA) and 4-hydroxynonenal (4-HNE), continuously accumulate during ferroptosis in cancer cells and facilitate cell membrane rupture and ferroptosis occurrence (Tang et al., 2021). Collectively, the above evidences suggest that PUFAs peroxidation is a vital prerequisite for ferroptosis. Hence, effective regulation of lipid peroxidation can help application of ferroptosis in cancer treatment.

### 3.3 Antioxidant defense system

Ferroptosis is a complex process interwoven with the imbalance between iron metabolism, lipid peroxidation and antioxidant defense in cells. To cope with oxidative damage, cancer cells have evolved the strengthened antioxidant capacities with the distinct defense pathways to evade ferroptosis and support tumor development. Thus, ferroptosis inducers can be a powerful strategy for cancer therapy, including the inhibitors of key defense pathways in ferroptosis. Here, it is focused to investigate the role of these associated signaling pathways that inhibit ferroptosis.

#### 3.3.1 SLC7A11/ GSH/ GPX4 signaling pathway

The SLC7A11/GSH/GPX4 pathway is a classical and central antioxidant system in ferroptosis (Li et al., 2022). Among them, GPX4 stands out as a principal regulator of ferroptosis. SLC7A11 (also named xCT) is the transporter subunit of the sodium-dependent cystine-glutamate antiporter System xc⁻, decisive for the exchanges of intracellular glutamate for extracellular cystine (Liu et al., 2020). SLC7A11 mediates the synthesize of GSH from condensation of glutamate, cystine, and glycine (Tang et al., 2021). As the crucial upstream node of SLC7A11/GSH/GPX4 signaling pathway, the inhibition of SLC7A11 leads to impaired antioxidant capacity, depleted GSH, inactivated GPX4, increased lipid ROS, ultimately induces ferroptosis in cancer cells (Jiang et al, 2021). GSH is not only a core antioxidant, but also the main cofactor of GPX4 (Zhang et al., 2023). Suppressing the synthesis and utilization of GSH facilitate ferroptosis onset, and furthermore, the function of GSH in ferroptosis is dependent on GPX4. As a central inhibitor of ferroptosis, GPX4 utilizes GSH to moduate ferroptosis through inhibiting lipid hydroperoxides. Moreover, GPX4 degradation can also result in ferroptotic cell death. In addition, the subcellular localization of GPX4 contributes to its distinct roles in cell protection from ferroptosis, representing cytosolic GPX4, rather than mitochondrial GPX4, has a crucial role in ferroptosis resistance. Thus, modulation of these GPX4-dependent antioxidants is essential to promote ferroptosis for cancer prevention and therapy.

#### 3.3.2 Keap1/Nrf2/HO-1 signaling pathway

In cancer microenvironment with ROS accumulation and chronic inflammatory responses, Nuclear factor erythroid 2-related factor 2 (Nrf2)-mediated antioxidant defense is highly required for cancer cells survival. Especially, the Kelch-like ECH-associated protein (Keap1) /Nrf2/HO-1 signaling pathway plays a central regulatory role in maintaining homeostasis (Liu et al., 2023). KEAP1, a ubiquitinated enzyme, binds to Nrf2 and facilitates its degradation, negatively regulates Nrf2, thereby sustain cancer development (Dodson et al., 2019). Nrf2, a master regulator of cellular antioxidant responses, triggers transcriptional response for ferroptosis suppression. Nrf2 serves as the primary transcriptional regulator of genes such as SLC7A11, GPX4, HO-1, Keap1 and Nrf2. Inhibition of Nrf2 results in downregulated SLC7A11 expression, reduced GSH synthesis, impaired GPX4 activity, accumulated lipid peroxides, ultimately induction of ferroptosis (Yuan et al., 2021). HO-1 is positively regulated by Nrf2 (Yan et al., 2023). Upregulation of Nrf2 promotes HO-1 transcription, contributing to the balance of cellular redox (Yang et al., 2021). Under resting condition, Nrf2 activity is inhibited by KEAP1 and maintained at a basal level. In contrast, under oxidative stress, KEAP1 releases abundant Nrf2 and subsequently Nrf2 transactivates HO-1, functioning in cytoprotective defense (Chiang et al., 2018). Therefore, targeting the Keap1/Nrf2/HO-1 axis may be promising to sustain antioxidant stress and enhance therapeutic effects in cancer cells.

#### 3.3.3 GPX4-independent ferroptosis pathways

In addition to GPX4-dependent antioxidant system in ferroptosis, so far there are alternative identified ferroptosis pathways, including ferroptosis suppressor protein 1(FSP1)/coenzyme Q10 (Co Q10) (Dai et al., 2024), dihydroorotate dehydrogenase (DHODH) /CoQH2 (Mao et al., 2021), GTP cyclohydrolase 1 (GCH1)/ tetrahydrobiopterin (BH4) (Kraft et al., 2020), 7-dehydrocholesterol (7-DHC) (Cao et al., 2025), and phospholipid modifying enzymes MBOAT1/2 (Liang et al., 2023), which act as suppressing ferroptosis wither by scavenging radicals for lipid peroxidation or modulating genes regulating lipid peroxidation. FSP1, as the GSH-independent coenzyme Q oxidoreductase in extramitochondrial membranes, has been identified to act as parallel with GPX4, representing another pivotal regulatory mechanism for ferroptosis resistance (Li et al., 2023). Mechanistically, FSP1 catalyzes the regeneration of CoQ10 (ubiquinone) through /nicotinamide adenine dinucleotide phosphate (NADPH) to CoQ10H2 (ubiquinol), and CoQ10H2 act as a lipophilic antioxidant to trap free radicals to suppress lipid peroxidation in the cell membrane, finally inhibit ferroptosis. Meanwhile, FSP1 also exhibits oxidoreductase activity linked to ferroptosis through mediating vitamin K (VK) to VKH2. In addition, Liver cancer is more dependent on FSP1 overexpression mediated by Keap1/Nrf2 to inhibit ferroptosis, and using FSP1 inhibitor iFSP1, FSP1 effectively induces ferroptotic cell death in liver cancer and profoundly increased immune infiltrates to unleash anti-cancer immune response (Cheu et al., 2023). Recently, it is reported that phase separation of FSP1 required N-terminal myristoylation, specific amino acid residues (S187, L217 and Q319), induces lipid peroxidation and ferroptosis (Nakamura et al., 2023). DHODH is a flavin-dependent mitochondrial enzyme in pyrimidine synthesis. In contrast to FSP1 function in extramitochondrial membranes, DHODH has been identified as a mitochondrial suppressor of ferroptosis through reducing CoQ10 to block lipid peroxidation in mitochondria. DHODH inhibition can also influence changes of GPX4 in mitochondria. Interestingly, DHODH and GPX4 constitute two primary defenses to detoxify lipid peroxidation in the mitochondria, and inhibiting one pathway can enforce cell more partial to the other one, whereas inactivation of both can lead to ferroptosis triggered by mitochondrial lipid peroxidation (Mao et al., 2021). GCH1 is a rate-limit enzyme of BH4 synthesis, which functions as an antioxidant in parallel with CoQ10 to block lipid peroxidation and ferroptosis. GCH1 can remodel lipid environment in plasma membranes to promote the accumulation of reduced CoQ10 and deplete FUPAs, ultimately increase the sensitivity to ferroptosis (Kraft et al., 2020). These pathways compensate for the survival benefits when the classic GPX4-dependent system is impaired. These also reveals that ferroptosis antioxidant systems might vary based on different subcellular compartments, thereby highlighting the new direction to explore ferroptosis regulation in different organelles.

### 3.4 Other regulatory mechanisms of ferroptosis

There are several classical signaling pathways, which either synergize with ferroptosis or act as upstream regulatory signals that influence cancer cell survival and proliferation, including PI3K/Akt, Wnt/β-catenin, p53, ACSL4, Caspase-1-dependent pyroptosis, and AMP-activated protein kinase (AMPK)/ Beclin1 (BECN1) signal pathways. Specially, the pro-survival pathway PI3K/Akt directly affects the expression of key ferroptosis-related proteins, such as GPX4, SLC7A11, Nrf2, and ACSL4, thereby affecting ferroptotic metabolism (Su et al, 2024; Wang et al, 2024a). The Wnt/β-catenin pathway benefits ferroptosis by promoting β-catenin binding to TCF/LEF transcription factors, which in turn reduces GPX4 activity and increases lipid peroxidation (Wang et al., 2022). In addition, the cancer suppressor p53 downregulates SLC7A11 expression to inhibit System Xc⁻ function, reduce GSH synthesis, finally increase cellular sensitivity to oxidative stress, which is not required GPX4 (Stockwell et al, 2020). As a pivotal enzyme in lipid metabolism, ACSL4 activates PUFAs, metabolize AA int AA-CoA, and sensitizes cancer cells to lipid peroxidation and ferroptosis (Ding et al., 2023). Furthermore, ACSL4-dependent ferroptosis mechanism is associated with metabolic lipid reprogramming, and T cell infiltration, indicating the crosstalk between immune system and lipid metabolism (Liao et al., 2022). Coordination between ferroptosis and Capase-1-dependent pyroptosis markedly reduces cancer tolerance and improve treatment efficacy (Wan et al, 2023). Furthermore, AMPK, inhibits SLC7A11 activity and GSH synthesis through mediating phosphorylated BECN1, further facilitates ferroptosis in cancer cells (Chen et al, 2021). These findings reflect the complexity of ferroptosis regulation involving different molecular pathways.

## 4 Effects of plant polysaccharides on cancer treatment via ferroptosis

**FIGURE 2:**
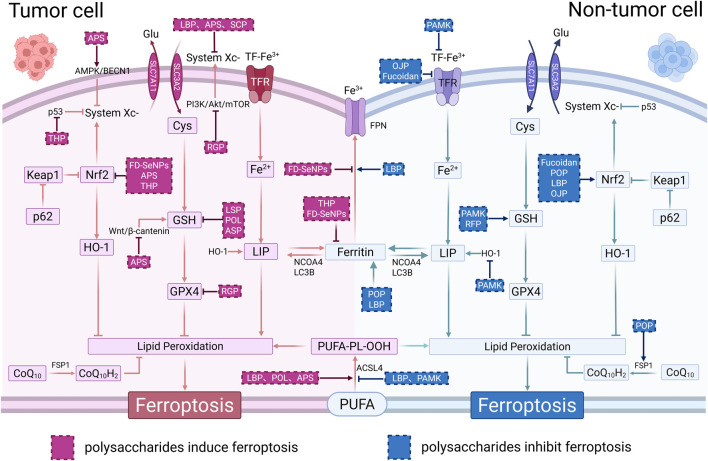
Anticancer mechanisms of plant polysaccharides through regulation of ferroptosis. The left pink section illustrates the role of plant polysaccharides as ferroptosis inducers in cancer cells. The right blue section depicts their role as ferroptosis inhibitors in non-cancer cells, protecting normal tissues from damage during anticancer treatment.

Polysaccharides possess significant anticancer potential by inhibiting cancer cell growth, regulating immunity, improving the cancer microenvironment, and overcoming drug resistance. In last decade, polysaccharides have exhibited regulatory activity in cancer through ferroptosis, which make them novel therapeutic strategies in cancer treatment. This review evaluated several plant polysaccharides that have been proven to exert potential antitumor effects through ferroptosis, showing promise as novel candidate drugs (Table 1). Figure 2 summarized the main mechanisms of plant polysaccharides in cancers targeting ferroptosis.

**TABLE 1:** Molecules and mechanisms of plant polysaccharides in cancers targeting ferroptosis.

Polysaccharide	Main constituents and ratio and MW	Source	Dose and treat time	Model	Ferroptosis pathway	Altered factors and pathways	Reference
*Lycium barbarum* polysaccharide (LBP)	Arabinose, glucose, galactose, mannose, xylose and rhamnose; NA; NA	The fruit of *Lycium barbarum L.*	4 and 6 mg/mL for 48 h	MCF-7, MDA-MB-231, breast cancer cell line	xCT/GSH/GPX4 pathway	xCT↓, GSH↓, GPX4↓, MDA↑, ROS↑, Fe^2+^↑	Du et al. (2022)
Red ginseng polysaccharide (RGP)	Glucose, galactose, arabinose, mannose, xylose and rhamnose; NA; NA	*Panax ginseng* C.A.Mey.	50, 100, 200, 400, 800 and 1,600 μg/mL for 0, 12, 24 and 48 h.	A549 lung cancer cell line. MDA MB-231 breast cancer cell line	GPX4 pathway	GPX4↓, LDH↑, ROS↑	Zhai et al. (2022)
*Astragalus* polysaccharide (APS)	NA	The root of *Astragalus mongholicus Bunge*	100 mg/mL for 48 h	Caov-3, SKOV3 ovarian cancer cell line	Nrf2/SLC7A11/GPX4 pathway	ROS↑, MDA↑, Fe^2+^↑, GSH↓, Nrf2↓, SLC7A11↓, GPX4↓	Zhang et al. (2024)
*Portulaca oleracea L.* polysaccharide (POL)	Glucose, galactose, arabinose, rhamnose; NA; NA	*Portulaca oleracea L.*	(*in vitro*) 50 and 100 μg/mL for 48 h (*in vivo*) 50 mg/kg for 3 weeks	SKOV3, Hey ovarian cancer cell line; nude mice	ACSL4 pathway	ROS↑, Fe^2+^↑, MDA↑, ACSL4↑, MMP↓, GSH↓	Xia et al. (2024)
*Astragalus* polysaccharide (APS)	NA	The root of *Astragalus mongholicus Bunge*	50, 100 and 200 μg/mL for 48 h	HepG2, hepatocellular carcinoma cell line	Wnt/β-catenin pathway	Wnt↓, β-catenin↓, GSH↓, GPX4↓, ROS↑, Fe^2+^↑, ACSL4↑, LPO↑	Zhu et al. (2025)
*Lycium barbarum* polysaccharide (LBP)	Arabinose, glucose, galactose, mannose, xylose, rhamnose; NA; NA	The fruit of *Lycium barbarum L.*	20, 40 and 60 μmol/L for 48 h	NOZ, SGC-996, gallbladder cancer cell line	Wnt/β-catenin pathway	Wnt3a↓, β-Catenin, Fe^2+^↑, ROS↑, MDA↑, ACSL4↑, GPX4↓	Xin et al. (2024)
*Angelica sinensis* polysaccharide (ASP)	Mannose, glucosamine, glucuronicacid, galacturonic-acid, glucose, galactose, arabinose, in the molar ratio of 2.53: 0.9: 1.18: 3.51: 82.03: 7: 2.86, mainly glucose	*Angelica sinensis (Oliv.) Diels*	(*in vitro*) 50, 100, 150, 200, 250 and 300 μg/mL for 48 h (*in vivo*) 0.2 mg/kg for 21 days	SKOV3, A2780, SKOV3/DDP and A2780/DDP ovarian cancer cell line; BALB/c nude mice	GSH/GPX4 pathway	Fe^2+^↑, MDA↑, ROS↑, SOD↓, GSH↓, GPX4↓	Guo et al. (2024)
*Lotus seedpod* polysaccharide (LSP)	Fucose, rhamnose, arabinose, glucose, galactose, mannose, fructose, galacturonicacid, glucuronic acid; in the molar ratio of 2.70: 1.02: 8.15: 45.63: 20.63: 1.44: 2.59: 16.4; MW 1240 KDa	*Lotus seedpod*	300, 900 and 1,500 μg/mL for 12, 24 h	HepG2 hepatocellular carcinoma cell line	GSH/GPX4 pathway	GSH↓, GGCT↓, OPLAH↓, GCLC↓, GSS↓, G6PD↓, PGD↓, IDH1↓, GSR↓, GSSG↓, GSTA1↓, GSTM2↓, GSTM3↓, GSTM4↓, GSTT1↓, GSTO1↓, GPX4↓, PRDX6↓, ABCC2↓, SLC25A39↑, MDA↑, ROS↑, Fe^2+^↑	Lin (2024)
*Astragalus* polysaccharide (APS)	MW 301 kDa	The root of *Astragalus mongholicus Bunge*	(*in vitro*) 10, 20 mg/mL for 24, 48 h	A549 lung adenocarcinoma cell line	GSH/GPX4 pathway	ROS↑, MDA↑, GSH↓, GPX4↓	Lee et al. (2024)
*Tetrastigma hemsleyanum* polysaccharide (THP)	Mannose, glucuronic acid, rhamnose, galacturonic acid, glucose, galactose, Arabinose; NA; NA	*Tetrastigma hemsleyanum Diels & Gilg*	(*in vitro*) 15, 30, 60 μg/mL 24 h (*in vivo*) 150 mg/kg	MDA-MB-231, 4T1 triple negative breast cancer cell line	xCT/GSH/GPX4 pathway Nrf2/NCOA4/FTH1 pathway	Fe^2+^↑, MDA↑, GSH↓, ROS↑, xCT↓, GPX4↓, p53↑, NCOA4↑, Nrf2↓, FTH1↓	Shang et al. (2024)
*Schisandra* polysaccharide (SCP)	Mannose, rhamnose, galacturonic acid, galactose, Arabinose in the ratio of 0.52 : 0.58 : 5.11 :2.02 : 1.10; NA	Schisandra *chinensis (Turcz.) Baill.*	(*in vivo*) SCP: 100, 200,400 mg/kg for 7 days SCPN: 25, 200, 400 mg/kg 7 days	HepG2 hepatocellular carcinoma	xCT/GSH/GPX4 pathway Mitochondrial apoptosis pathway Caspase-1-dependent pyroptosis pathway·	IL-6↑, TNF-α↑, Cytochrome C↑, Bax↑, NLRP3↑, gasdermin D↑, Caspase-1↑, ROS↑, MDA↑, LDH↑, Fe↑, IL-10↓, GSH↓, SOD↓, SLC7A11↓, GPX4↓	Wang (2024)
Red ginseng polysaccharide (RGP)	Glucose, galactose, arabinose, mannose, xylose and rhamnose; NA; NA	*Panax ginseng* C.A.Mey.	(*in vitro*) 50, 100, 200 μg/mL for 12, 24, 48 h (*in vivo*) 75, 150, 300 mg/kg for 7 days	RGM-1, AGS, gastric cancer cell line	PI3K/Akt pathway	ROS↑, Fe^2+^↑, MDA↑, LDH↑, SLC7A11↓, GPX4↓, p-PI3K↓, p-Akt↓, p-PI3K/PI3K↓, p-Akt/Akt↓	Wang et al. (2024a)
Hydrophilic angelica polysaccharide (ASP)	Mannose, glucosamine, glucuronicacid, galacturonic-acid, glucose, galactose, arabinose, in the molar ratio of 2.53: 0.9: 1.18: 3.51: 82.03: 7: 2.86	*Anethum graveolens L.*	2, 4, 6, 12, 24 μg/mL for 24 h	HepG2 hepatocellular carcinoma cell line	GSH pathway	GSH↓	
Fucoidan-selenium nanoparticle (FD-SeNPs)	L-fucose, galactose, xylose, glucuronic acid, mannose, glucose, arabinose; NA; NA (purity 97%)	*Fucus vesiculosu*	500 μg/mL for 48 h	HepG2 hepatocellular carcinoma cell line	Nrf2/HO-1 pathway, SLC7A11/GSH/GPX4 pathway	ROS↑, MDA↑, Fe^2+^↑, Ferritin↑, GSH↓, Nrf2↓, HO-1↓, SLC7A11↓, GCLC↓, GPX4↓, SLC40A1↓	Chen et al. (2025)
Polysaccharide of *Atractylodes macrocephala* koidz (PAMK)	Glucose, galactose, rhamnose, arabinose, mannose, galacturonic acid, xylose; NA; MW 2.1∼220 kDa	*Atractylodes macrocephala*	400 mg/kg in diets for 28 days	Goslings	GSH/GPX4 pathway	ROS↓, MDA↓, Fe^2+^↓, GSH↑, GPX4↑, ACSL4 ↓, COX-2 ↓, TFR1↓, VCAD2/3↓, TF↓	Zhou et al. (2022)
*Schisandra* polysaccharide (SCP)	Mannose, rhamnose, galacturonic acid, galactose, arabinose; in the molar ratio of 0.52:0.58:5.11: 2.02: 1.10; NA	*Schisandra chinensis (Turcz.) Baill.*	100 μL medicated serum from 100, 200, 400 mg/kg	HepG2 hepatocellular carcinoma cell line	xCT/SLC7A11/GPX4 pathway	GSH↓, SOD↓, MDA↑, Fe^2+^↑, ROS↑, LDH↑, xCT↓, SLC7A11↓, GPX4↓	Wang (2024)
Fucoidan	L-fucose, galactose, xylose, glucuronic acid, mannose, glucose, arabinose; NA; NA (purity 97%)	*Fucus vesiculosu*	200 mg/kg for 12 days	C57BL/6 mice	Nrf2/GSH/GPX4 pathway	GPX4↑, Nrf2↑, GSH↑, TfR1↓, FTH1↑, Ptgs2↓, ROS↓, MDA↓	Wang et al. (2024b)
*Ophiopogon japonicus* polysaccharide (OJP)	Glucose, fructose, galactose, arabinose, mannose; NA; NA (purity, 98.50%)	*Ophiopogon japonicus (Thunb.) Ker Gawl.*	50, 100, 200 μg/mL for 24 h	AC16 human cardiomyocyte cell line	Nrf2/GPX4 pathway	LDH↓, CK-MB↓, cTnI↓, MDA↓, ROS↓, TFR1↓, Fe^2+^↓, GPX4↑, ATP↑, MMT↑, Nrf2↑	Chen et al. (2025)
*Polygonatum odoratum* polysaccharide (POP)	Fructose, galactose, rhamnose, mannose, glucose, xylose, galacturonic acid; NA; NA	*Polygonatum odoratum (Mill.) Druce*	400 mg/kg for 9 days	C57BL/6j mice	Nrf2/HO-1 pathway	Scr↓, BUN↓, MDA↓, GSH↑, FSP1↑, FTH1↑, GPX4↑, Nrf2↑, HO-1↑	Jiang et al. (2024)
Lateral root polysaccharides of *Aconitum* *carmichaelii* (RFP)	Rhamnose, D-galacturonic acid, D-glucose, D-galactose, xylose, L-arabinose; in the molar ratio of 1: 2.34: 59.12: 4.64: 1.88: 10.72; MW 7.973–1,211.136 kDa	*Aconitum carmichaelii Debeaux*	(*in vitro*) 5,000,2500, 1,250, 625, 312 μg/mL for 24 h (*in vivo*) 200, 400, 800 mg/mL for 10 days	NRK-52E rat renal tubular epithelial cell line; male Kunming mice	GSH/GPX4 pathway	Fe^2+^↓, MDA↓, SOD↓, GSH↑, GPX4↑, 4-HNE↓	Tian et al. (2022)
*Lycium barbarum* polysaccharide-glycoprotein (LBP)	Galactose, rhamnose, arabinose, glucose, mannose, xylose; NA; MW 10–200 kDa	*The fruit of Lycium barbarum L.*	(*in vitro*) 0.8 μg/mL for 72 h (*in vivo*) 3 g/100 mL for 9 days	Human keratinocyte HaCaT cells Sprague–Dawley rats	Nrf2/HO-1 pathway	ROS↓, LPO↓, Fe^2+^↓, ACSL4↓, PTGS2↓, GSH↑, Nrf2↑, GCLM↑, GCLC↑, HO-1↑, NQO1↑, FTH1↑, FTL↑, SLC7A11↑, SCL40A1↑	Jiang et al. (2023)

### 4.1 Inhibition of cancer cell growth

Plant polysaccharides have cytotoxic and inhibitory effects on cancer cells, which is related with ferroptosis. Down regulation of GPX4 pathway is involved in polysaccharides-induced inhibition in cancer cells. *Lycium barbarum* polysaccharide (LBP) altered mitochondrial structure and function and promoted ferroptotic breast cancer death at 4, 6 mg/mL via the xCT/GPX4 pathway (Du et al., 2022). Red ginseng polysaccharide (RGP) induced ferroptosis in lung cancer cells and breast cancer at the concentration range of 50 to 1600 µg/mL by inhibiting GPX4 expression. GPX4 overexpression blocked RGP-induced GPX4 downregulation and restored LDH and ROS accumulation, conferred cell resistance to drug (Zhai et al., 2022). Plant polysaccharides also can inhibit upstream transcriptional factor Nrf2 expression, to suppress its downstream target proteins including HO-1, SLC7A11, and GPX4, ultimately leading to cancer cell death (Wang et al., 2024b). Astragalus polysaccharide (APS) induced ferroptosis in ovarian cancer at 100 mg/mL through inhibiting the Nrf2/SLC7A11/GPX4 pathway (Zhang et al., 2024). Furthermore, plant polysaccharides can promote excess lipid peroxidation for inhibition of cancer cell. *Portulaca oleracea L*. polysaccharide (POL) sustained ovarian cancer progress by upregulating ACSL4-mediating ferroptosis in vitro and in vivo, correspondingly, knockdown of ACSL4 significantly reversed POL-induced ferroptosis in ovarian cancer cells, and this form of cell death was remarkably rescued by Ferrostatin-1 and deferoxamine (Xia et al., 2024). In addition, plant polysaccharides also induce ferroptotic cancer cells by inactivating signaling pathways closely related to cell proliferation, thereby suppressing cancer growth. Astragalus polysaccharides (APS) inhibited the proliferation and invasion of hepatocellular carcinoma at the concentration range of 50 to 200 μg/mL by suppressing the Wnt/β-catenin pathway along with reduced GSH content and depleted GPX4 activity, and increased ACSL4, ROS, LPO and Fe^2+^ levels (Zhu et al., 2025). *Lycium barbarum* polysaccharide (LBP) also enhanced ferroptosis and inhibited the proliferation of gallbladder cancer at 40 μmol/L via inactivation of Wnt/β-catenin pathway (Xin et al., 2024). These studies revealed that regulation of plant polysaccharides in cancer cell survival and proliferation is mainly dependent on classical ferroptotic mechanism, including GPX4-dependent, Nrf2-mediated, accumulation of lipid peroxidation. Notably, the results of these studies above showed that the inhibitory concentrations of LBP, APS, RGP and POL were within the broad dosage ranges of 50 μg/mL to 100 mg/mL in various cancer cells for 48 h, especially APS and LBP, exhibiting various therapeutic effects. This variation might be due to the differences in the source, manufacture and purity of these polysaccharides. The structure and composition of polysaccharides vary largely dependent on their sources, which profoundly influences their subsequent pharmacodynamics. The lack of standard extraction and purification protocols greatly constrain the monosaccharide composition and purity of these polysaccharides, ultimately leading to the unstable experiment results. Additionally, the inhibitory effects of these polysaccharides used exhibited a dose-dependent manner and further at a relative higher concentration, to 100 mg/mL for APS. This is noteworthy that the underlying toxicity of polysaccharides caused by the excessive concentration used and its damage on cells, which may constrain the application of polysaccharides.

### 4.2 Reduction of chemotherapy resistance

Drug resistance of cancer chemotherapy represents a severe clinical challenge that affects therapeutic efficacy and prognosis. Activation of ferroptosis would be helpful to enhance sensitivity of cancer cells to chemotherapeutic agents (Zheng and Guan, 2023). Unlike synthetic compounds, plant polysaccharides can exhibit high biosafety while inhibit drug resistance in cancer therapy. As a commonly used clinic chemotherapeutic agent, cisplatin resistance in cancer is associated with the decreased intracellular drug accumulation, and the enhanced DNA repair capacity along with dysregulation of ferroptosis-related signaling pathways. In contrast, several polysaccharides have been proven to reduce cisplatin resistance (Guo et al., 2024). *Angelica sinensis* polysaccharide (ASP) combined with cisplatin increased ferroptosis in cisplatin-resistant ovarian cancer cells through inhibition of GPX4 expression with significant GSH depletion and no obvious toxicity to liver, kidney, and cardiac tissues, ultimately reversed the resistance to cisplatin (Guo et al., 2024). Similarly, *Lotus seedpod* polysaccharides (LSP) combined with cisplatin induced ferroptosis in hepatocellular carcinoma via GSH-dependent pathway (Lin, 2024). These findings demonstrate that polysaccharides combination with cisplatin has more effective effects on cancer cells through regulation of ferroptosis pathways than any one alone. Furthermore, polysaccharides not only enhance cancer cell sensitivity to cisplatin, but also improve the bioavailability of cisplatin in cancer cells. This enhanced effect might be associated with polysaccharide-induced intracellular GSH decrease, intensifying drug accumulation in cancer cells to function with significant anticancer activity. It is reported that inducing ferroptosis by inhibiting Nrf2/Keap1/xCT signaling readily sensitized cisplatin-resistant cells to cisplatin in gastric cancer (Fu et al., 2021). Although there are few sufficient experimental or clinical evidences about the synergy or addition effect of polysaccharides in cancer treatment, these findings certainly highlight the advantage of polysaccharides in conjunction with chemotherapy or radiotherapy, and also represent a novel strategy for addressing chemoresistance in cancer therapy.

### 4.3 Enhancement of drug sensitivity

Plant polysaccharides can enhance cancer cell sensitivity to anticancer drugs and improve therapeutic efficacy. Under normoxic conditions, most cancers rely on glycolysis for energy production. Metformin, involved in the metabolic reprogramming of tumor cells, can downregulate glycolysis and inhibit cancer growth, but its therapeutic efficacy is limited in hypoxic microenvironments (Granja et al., 2015). Astragalus polysaccharide (APS) combined with metformin exhibited a collateral effect on lung cancer to enhanced lung cancer cell sensitivity to metformin via inactivated GPX4/GSH ferroptosis pathway (Lee et al., 2024). The increased efficacy might be due to their collaboration in modulation of immunity and metabolism in lung cancer cells, and moreover, APS involvement reduced the required dosage of metformin in cancer cells. Additionally, as one clinical drug used to inhibit cancer development, selenite combined with LSP regulated the GPX4/GSH pathway to promote ferroptosis in hepatocellular carcinoma and enhanced hepatocellular carcinoma cell sensitivity to selenite (Lin, 2024). These observations demonstrate that these polysaccharides help to enhance the efficacy of chemotherapeutic agents in cancer cells through ferroptosis regulation and meanwhile decrease the drug dosage used to alleviate the toxicity to cells, which might be due to enhancement effects of polysaccharides in drug bioavailability. The in-depth mechanism is necessary to explore in future research.

### 4.4 Autophagy-mediated ferroptosis

Plant polysaccharides can enhance ferroptosis against cancer by activating autophagy pathways, causing ferritin degradation and Fe^2+^ accumulation. AMPK phosphorylates BECN1 to promote autophagy, thereby inhibiting xCT activity, reducing GPX4 expression, and finally leading to ferroptosis (Song et al., 2018). APS induced GPX4-dependent ferroptosis in urothelial carcinoma by activating the AMPK/BECN1-mediated autophagy pathway and promoting the formation of the BECN1-xCT complex (Tong et al., 2023). *Tetrastigma hemsleyanum* polysaccharide (THP) combined with doxorubicin induced ferroptosis and autophagy in triple-negative breast cancer through the Nrf2/NCOA4/FTH1 and xCT/GSH/GPX4 pathway (Shang et al., 2024). These findings clarified that autophagy is involved in ferroptosis induced by polysaccharides, and enhance ferroptosis effects through autophagy activation, thereby improving its anticancer therapeutic efficacy. Recent discoveries showed that lipid peroxidation and its products function at various stages of autophagy, influencing autophagosome formation of and downstream proteins recruitment (Wang et al., 2023). Excessive autophagy induces ferroptosis through increased lipid peroxidation, and lipid peroxidation is one specific hallmark of ferroptosis, which suggest autophagy and ferroptosis interact with each other. Ferritinophagy mediated by NCOA4, is at the intersection of ferroptosis and autophagy, and have been confirmed to induce ferroptosis in various cancer cell lines (Sun et al., 2022).

### 4.5 Combined regulation of ferroptosis, apoptosis and pyroptosis

Other than ferroptosis, plant polysaccharides can also trigger apoptosis and pyroptosis. LSP effectively stimulated apoptosis in HepG2 cells in a dose-dependent manner, along with specific ferroptotic observations including the collapsed mitochondrial membrane potential, accumulation of MDA and ROS, increased Fe2+ levels (Lin, 2024). Ferroptosis results from abnormal increased ROS levels that leads to lipid peroxidation, meanwhile the products of lipid peroxidation interact with membrane receptors and transcription factors/repressors to activate intrinsic and extrinsic signaling pathways for apoptosis (Elkin et al., 2018). These implied that polysaccharides may activate numerous different cell death forms through initial modulation of lipid peroxidation to collateral regulation of signaling pathways, thereby controlling the decision to die. Of note, mitochondria function might also be involved in regulation of polysaccharides in apoptosis and ferroptosis. Mitochondrial functions influence lipid homeostasis and the extent of lipid peroxidation for ferroptosis, as well as maintain mitochondrial membrane integrity and protect release of intermembrane space protein for apoptosis (Khatun et al., 2024).

Furthermore, the PI3K/Akt pathway plays a crucial role in polysaccharides-induced ferroptosis. Targeted inhibition of PI3K/Akt pathway provides a probable option for cancer treatment. RGP reduced AQP3 expression to inhibit the PI3K/Akt pathway, thereby promoting ACSL4-mediated ferroptosis in gastric cancer cells (Wang et al., 2024a). Schisandra polysaccharides (SCP) simultaneously induced the mitochondrial apoptosis pathway, Caspase-1-dependent classical pyroptosis pathway, and SLC7A11/GSH/GPX4 ferroptosis pathway, exerting anti-hepatocellular carcinoma effects (Wang, 2024). These studies indicate that multiple pathways and multiple mechanisms are involved in plant polysaccharides against cancers, also reflect that the complexity of regulation network of polysaccharides in cell death.

### 4.6 Application of nano delivery system

As plant polysaccharides possess biodegradability, biocompatibility, and inherent pharmacological activity, nanomaterials derived from plant polysaccharides exhibit significant advantages in improving drug utilization, pharmacokinetics, reducing toxicity, controlling drug release, and minimizing required dosages (Ni et al., 2024). AAAF@Cur nanoparticles, constructed with hydrophilic angelica polysaccharide (ASP) as a carrier, accelerated curcumin release under hypoxic conditions, concentration-dependently reduced GSH levels, and induced ferroptosis in HepG2 cells. Remarkably, AAAF@Cur enhanced specific uptake by HepG2 cells through ASGPR-mediated endocytosis without significant damage on normal cells (Liu et al., 2022). Fucoidan-selenium nanoparticles (FD-SeNPs) induced ferroptosis in hepatocellular carcinoma cells through downregulation of the Nrf2/HO-1 and SLC7A11/GSH/GPX4 pathways, characterized by elevated ROS, MDA, and Fe^2+^ levels, reduced GSH, mitochondrial shrinkage and cristae disruption, upregulation of ferroportin, and downregulation of SLC40A1 and Ferritin, ultimately disrupting iron homeostasis (Chen et al., 2025a). These data above showed that TCM polysaccharide nanoparticles utilize their targeting properties to improve bioavailability and bioactivity, influence the cancer microenvironment, reduce antioxidant capacity, regulate iron homeostasis, disrupt mitochondrial function, and induce cancer cell ferroptosis. These findings highlight their promise as a strategic direction for future cancer therapies.

### 4.7 Stimulation of immune response against cancers

Polysaccharides possess notable properties of immunomodulatory and anticancer, making them valuable therapeutic agents. Disorders in iron accumulation, ROS production and antioxidant systems damage the morphological structure and function of immune organs, thereby alter immune responses, and even contribute to disease progression (Li et al., 2022). Polysaccharide of *Atractylodes macrocephala Koidz* (PAMK) regulated the GPX4-dependent ferroptosis pathway to alleviate cyclophosphamide-induced thymic damage and improve immune function (Zhou et al., 2022). Furthermore, enhanced immune responses can in turn promote cancer ferroptosis. Recent research have showed that CD8^+^ T cells could enhance T cell–mediated immunity and release IFN-γ, downregulate SLC7A11 to lead to ferroptotic cancer cell death (Cai et al., 2023). *Tetrastigma hemsleyanum* polysaccharide (THP) regulated ferritin autophagy and ferroptosis in triple-negative breast cancer and improved the cancer microenvironment and immune function through increased CD4+ and CD8+ T cells as well as reduced regulatory T cells (Shang et al., 2024). Schisandra polysaccharides (SCP) regulated the SLC7A11/GSH/GPX4 ferroptosis pathway in HepG2 while altered the expression levels of cytokines including increased IL-6 and TNF-α levels, reducing IL-10 levels (Wang, 2024). These results demonstrated that polysaccharides can steer the imbalance of iron-dependent lipid peroxidation as one of the avenues to execute immunomodulation.

### 4.8 Alleviation of tissue injury by anticancer treatment

Although traditional cancer therapies serve as primary strategies for cancer treatment, their severe side effects including drug resistance, tissue damage, remarkably constraint the clinical application in cancer. Plant polysaccharides manifest the advantages of reversing cancer multidrug resistance, and lessening anticancer therapy-induced tissue injury. Doxorubicin (DOX), an antineoplastic drug widely used in anticancer treatment, can increase cardiotoxicity, lead to myocardial injury and heart failure (Wallace et al., 2020). Fucoidan regulated the Nrf2/GPX4 pathway to inhibit DOX-induced ferroptosis in myocardial tissue (Wang et al., 2024b). *Ophiopogon japonicus* polysaccharide (OJP) inhibited DOX-induced myocardial ferroptosis through activation of the Nrf2/GPX4 pathway, reduced LDH, CK-MB as well as cTn-I levels, and enhanced ATP generation, hence alleviated myocardial toxicity (Chen et al., 2025b). *Polygonatum odoratum* polysaccharides (POP) activated the Nrf2/HO-1 pathway to inhibit ferroptosis and alleviated cisplatin-induced acute kidney injury (Jiang et al., 2024). The lateral root polysaccharides of *Aconitum carmichaelii* (RFP) had the cytotoxicity effects on hydrogen peroxide-induced injury and prohibited apoptosis in vitro, by contrast, RFP attenuated cisplatin-induced acute kidney injury in vivo through inhibition of ferroptosis with the increased levels of GSH and GPX4, the decreased levels of MDA and 4-hydroxynonenal as well as the reduced lipid peroxidation (Tian et al., 2022). LBP regulated the Nrf2/HO-1 pathway to inhibit ferroptosis and reduce irradiation-induced oral mucosal damage, while Nrf2 knockdown eliminates protective effects of LBP (Jiang et al., 2023). These evidences demonstrated polysaccharides exert distinct functions on ferroptosis in cancer cells and non-cancer normal cells. Furthermore, polysaccharides have been identified as ferroptosis agonists and inhibitors, exhibiting anti-tumor efficacy by triggering ferroptosis and alleviating tissue injuries by inhibiting ferroptosis (Du et al., 2022; Dorschmann et al., 2021).

## 5 Conclusion and prospects

More and more attentions are attracted by plant polysaccharides due to their diverse biological effects including anticancer activity, enhancement of immune responses, antioxidation and stimulation of endogenous factor release. Extensive studies have demonstrated that plant polysaccharides play crucial roles in prevention and treatment of cancers by regulating ferroptosis through multiple targets and signaling pathways. The major ferroptosis-related pathways influenced by polysaccharides are the SLC7A11/GSH/GPX4 pathway, Keap1/Nrf2/HO-1 pathway, NCOA4-mediated ferritinophagy pathway, ACSL4-mediated lipid peroxidation pathway, and their upstream signal such as PI3K/Akt, Wnt/β-catenin, p53, and the AMPK/BECN1 autophagy pathway.

**FIGURE 3:**
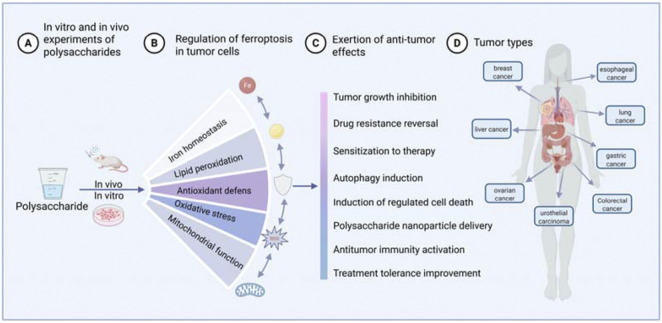
Anticancer effects of plant polysaccharides via ferroptosis both *in vitro* and *in vivo*.

Studies have demonstrated that plant polysaccharides regulate ferroptosis to exert anticancer effects *in vitro* and *in vivo* (Figure 3). First of all polysaccharides can act as ferroptosis inducers to suppress the growth of various solid cancers, including breast cancer, esophageal cancer, hepatocellular carcinoma, lung cancer, gastric cancer, ovarian cancer, colorectal cancer, and urothelial carcinoma, exhibiting favorable anticancer effects. In addition, their combination with conventional treatment strategies helps prevent drug resistance and enhances the body’s immunoregulatory capacity. Intriguingly, polysaccharides can alleviate anticancer drug-induced damage to normal tissues including the heart, liver, and kidneys, improving prognosis of cancer treatment. This dichotomy might be due to differential basal metabolism and redox status. Cancer cells have been shown to predispose to increased ferroptosis susceptibility due to characteristic iron-metabolism, constitutively higher ROS levels and targeted gene expression (Endale et al., 2023; Schwab et al., 2024). Cellular higher ROS levels decide the development of ferroptosis, and lipid peroxidation promote ferroptosis through enhancing lipotoxicity. This diversity of polysaccharide regulation in ferroptosis sheds light on their promising application in cancer treatment.

The activity of polysaccharides is closely related to their structure and composition. However, absence of identified source and standardized extraction and purification protocols have remarkably influenced the application of polysaccharides. Thereby, standardizing the source and extraction and purification procedure is one primary and critical step for polysaccharide toward enhancing their medicinal value.

Additionally, to circumvent the dose-dependent effects of plant polysaccharides in cancer treatment, the combination of nanocarriers with polysaccharides represents an emerging and promising strategy for cancer therapy, improving the cancer microenvironment, accelerating drug release, enhancing local drug concentrations in cancer tissues, significantly reducing toxicity, and strengthening therapeutic effects against cancers. Currently, research on the anticancer activity of plant polysaccharides primarily focuses on GPX4-dependent ferroptosis pathways, while GPX4-independent signaling pathways, such as FSP1/CoQ10, DHODH and GCH1/BH4 pathways, remain unexplored. In the future, more in-depth investigations are necessary to uncover the diverse ferroptosis-related mechanisms of plant polysaccharides.
